# Real time observation of the interaction between aluminium salts and sweat under microfluidic conditions

**DOI:** 10.1038/s41598-021-85691-8

**Published:** 2021-03-18

**Authors:** Yasine Sakhawoth, Jules Dupire, Fabien Leonforte, Marion Chardon, Fabrice Monti, Patrick Tabeling, Bernard Cabane, Robert Botet, Jean-Baptiste Galey

**Affiliations:** 1 L’Oréal Recherche and Innovation, 1 avenue Eugène Schueller, 93600 Aulnay-sous-Bois, France; 2 IPGG, MMN, 6 Rue Jean Calvin, 75005 Paris, France; 3 Université Paris-Saclay, CNRS, Laboratoire de Physique des Solides, UMR8502, 91405 Orsay, France; 4 LCMD, CNRS UMR8231, ESPCI, 10 rue Vauquelin, 75231 Paris cedex 05, France

**Keywords:** Self-assembly, Fluids, Gels and hydrogels, Chemical physics

## Abstract

Aluminium salts such as aluminium chlorohydrate (ACH) are the active ingredients of antiperspirant products. Their mechanism of action involves a temporary and superficial plugging of eccrine sweat pores at the skin surface. We developed a microfluidic system that allows the real time observation of the interactions between sweat and ACH in conditions mimicking physiological sweat flow and pore dimensions. Using artificial sweat containing bovine serum albumin as a model protein, we performed experiments under flowing conditions to demonstrate that pore clogging results from the aggregation of proteins by aluminium polycations at specific location in the sweat pore. Combining microfluidic experiments, confocal microscopy and numerical models helps to better understand the physical chemistry and mechanisms involved in pore plugging. The results show that plugging starts from the walls of sweat pores before expanding into the centre of the channel. The simulations aid in explaining the influence of ACH concentration as well as the impact of flow conditions on the localization of the plug. Altogether, these results outline the potential of both microfluidic confocal observations and numerical simulations at the single sweat pore level to understand why aluminium polycations are so efficient for sweat channel plugging.

## Introduction

Eccrine sweat glands are found in high density in human skin, with surface concentrations in the range of 100 glands per cm^2^ in the axilla region^1,2^. The major function of eccrine sweat glands found all over human body is to participate in body core temperature regulation by water evaporative heat dissipation under thermal stress conditions^3^, but they can also be stimulated by other conditions such as emotional stress or spicy food ingestion^4^. In the axilla region, sweat can be produced in large amounts leading to an unpleasant humidity sensation as well as sweat rings on clothes. Moreover, axillary humidity increases the development of body odours resulting from bacterial transformation of apocrine secretion^5^. Topically applied antiperspirants products have therefore been developed in order to limit the inconveniency of excessive axillary sweating. Aluminium salts are the only class of marketed compounds recognized as having clinical efficacy. It has been known for decades that their efficacy is associated with a superficial and temporary sweat pore plugging by aluminium and glycoproteins aggregates which consequently decreases the amount of sweat reaching the skin surface^6–8^. However, until now, there was a lack of mechanistic knowledge regarding this antiperspirant effect both at physicochemical and molecular levels.

**Figure 1:**
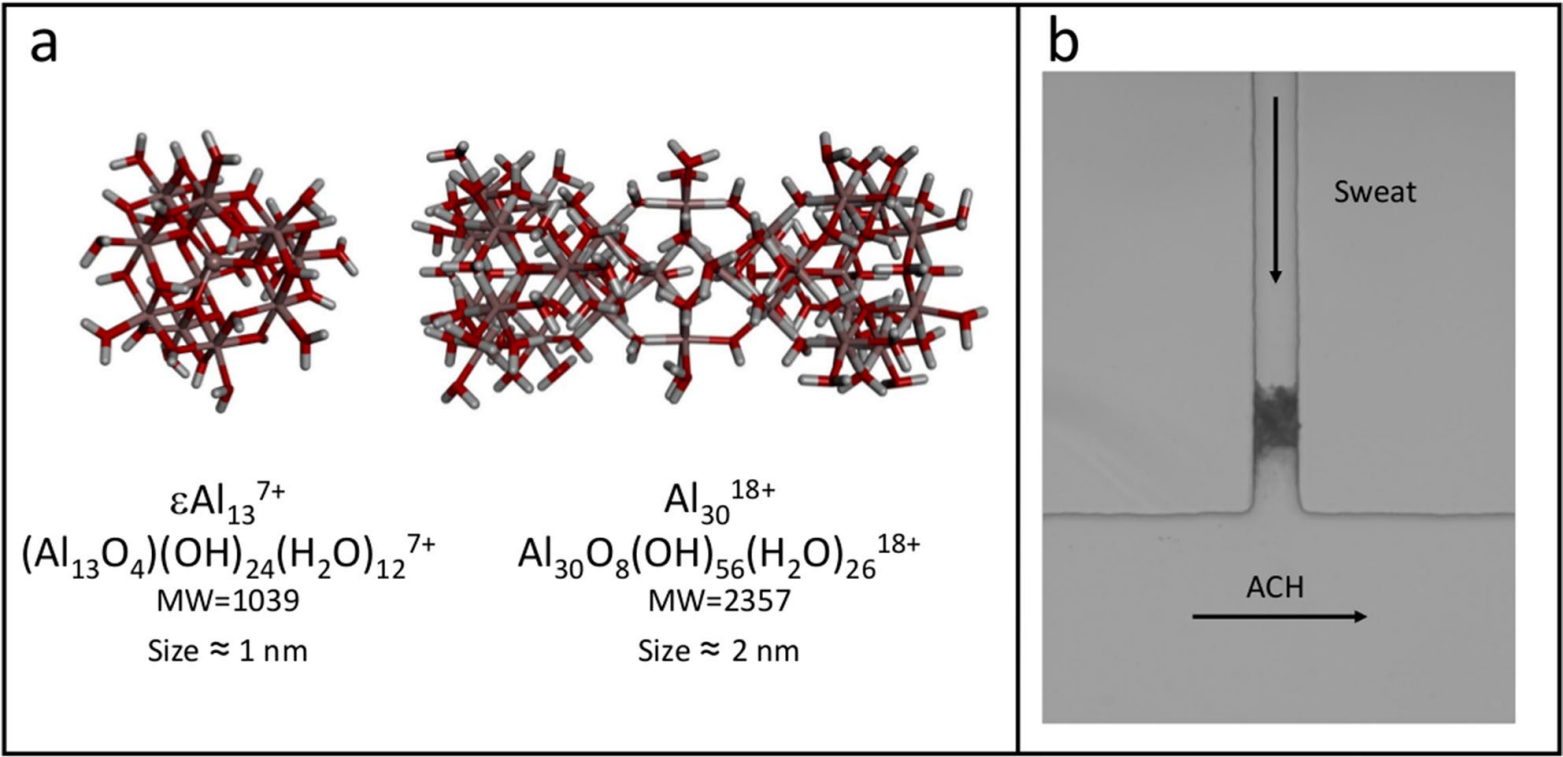
(**a**) Molecular modelling representation of εAl_13_ and Al_30_ clusters with corresponding nominal charge, molecular weight and size. These polycations are representative of active compounds present in the ACH solution. (**b**) Photograph of the microfluidic chip showing the two orthogonal channels mimicking a sweat pore and the skin surface. At the beginning of the experiment, the sweat channel (55 µm high × 50 µm wide) was flowed at 0.6 nL s^−1^ with natural human sweat collected from human volunteers. The ACH channel (55 µm high × 400 µm wide) was flowed at 60 nL s^−1^ ACH 15% (w/w) from left to right. The image shows the visual aspect of the plug after 30 min of pressure-controlled flow.

In aqueous systems, aluminium ions undergo hydrolysis and condensation resulting in a range of aluminium oxy-hydroxide clusters relevant to geochemical processes, but also to many applications ranging from coagulation for water treatment to antiperspirant activity^9–12^. Among the range of different aluminium salt specialties used for antiperspirant applications^13^, ACH (Aluminium Chloro Hydrate) is one of the most utilized forms. It is commercialized as a 50% concentrated solution pH (4–4.5) with a hydrolysis ratio close to 2.5. ACH solutions contain large polycationic species (from 1000 to more than 5000 Da), some of which have been fully characterized such as cage-like Keggin clusters^14^ εAl_13_ and Al_30_. These species bear very high net electric nominal charges of 7 + for εAl_13_ and 18 + for Al_30_ (Fig. 1a) and small size in the range 1–2 nm. Although net effective charges of εAl_13_ and Al_30_ are actually smaller, respectively 3.5 and 6.7 according to recent capillary electrophoresis studies^15^, they can produce strong electrostatic interactions with anionic polymers^16,17^. For example, most proteins naturally present in human sweat or constitutive of sweat pore walls are anionic at the pH of sweat^18^, and can thus aggregate in the presence of aluminium polycations. This electrostatic aggregation is considered to be the basis of the antiperspirant effect of ACH^19–22^.

In previous reports^21,23^, we have shown that the antiperspirant effect of ACH could be studied in dynamic conditions at the scale of a single sweat pore using a microfluidic model comprising two orthogonal PDMS channels connected by a T-junction. Microsyringe pumps delivered ACH solution in a channel, and sweat in the other one, at flow rates in the nL s^−1^ range, thereby mimicking physiological sweat flows^1^. This microfluidic model made it possible to observe the time-dependent clogging of channels of dimensions close to that of the sweat pores, in a dynamic environment where the roles of advective and diffusive flows can be studied. It can either be used with natural sweat collected from human volunteers or artificial sweat consisting of a saline solution containing a model protein such as Bovine Serum Albumin (BSA). It is assumed that the capacity of ACH to form plugs deeply inside sweat channel is related to its antiperspirant capacity^23^.

Small Angle X-ray Scattering (SAXS) experiments conducted under flowing conditions using BSA artificial sweat enabled us to demonstrate that the plugging of the sweat channels is linked to the aggregation of BSA by aluminium polycations diffusing against sweat flow and that the density of the aggregates increases exponentially over time thanks to BSA supply transported by the sweat flow^21^. However, these results were obtained with a model sweat containing 1% BSA, i.e. high protein concentration compared to natural sweat, and with microfluidic chips equipped with channels larger than real ones, *i.e.* 500 µm instead of 20–60 µm^24^. Moreover, these experiments were conducted at constant flow rates which are not representative of sweat secretion by eccrine sweat glands. Finally, a growth nucleation mechanism from channel walls was suggested but not directly demonstrated.

In the present paper, we confirm previous results under more physiological conditions, i.e. with 50 µm channels and pressure controlled flow rates, using either natural sweat or artificial sweat containing different proteins at low concentrations. Moreover, a range of physicochemical conditions was explored in order to determine the factors that influence channel clogging.

The identification of the clogging mechanisms was achieved using three complementary techniques:

- Confocal microscopy using fluorescein-labelled BSA gives images at controlled depths inside the sweat channel.
- Small angle X-ray Scattering (SAXS) gives detailed information on the amount and spatial arrangement of the molecules forming the plug.
- Numerical simulations can reproduce plug structures and provide information on the mechanisms leading to plug formation.

## Results and discussion

### Plug formation under physiological conditions

Microfluidic experiments were conducted using sweat channels with rectangular section (50 × 55 µm) and pressure controlled flow rates using either natural sweat or artificial sweat. ACH was flowed through a perpendicular channel with a height of 55 µm and a width of 400 µm; the latter channel figuring as an infinite reservoir. Figure 1b illustrates the plug formation inside the sweat channel using natural human sweat under these conditions. Plugging was achieved with a 15% (w/w) aqueous solution of ACH; 15% is a typical concentration found in common antiperspirant products. It corresponds to 50 mM Al_30_ if all polycations are in the form of Al_30_.

**Figure 2:**
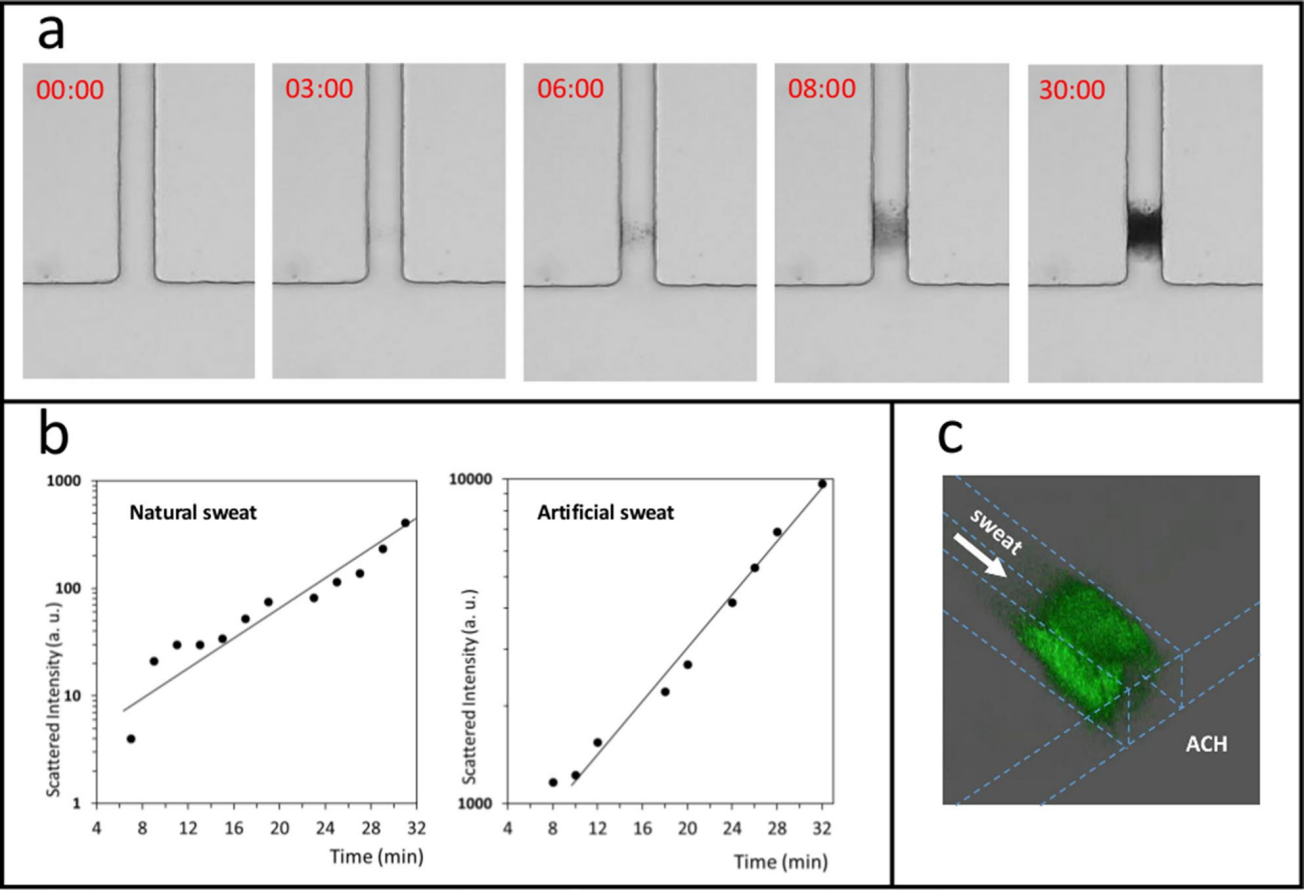
(**a**) Photographs of plug formation within the sweat channel at different times (in min) after contact of the two fluids, namely: artificial sweat, flowed at 0.6 nL s^−1^ at the beginning of the experiment and maintained at a constant pressure in the sweat channel, and an aqueous solution of ACH 15% flowed at 60 nL s^−1^ in the ACH channel. Artificial sweat composition (w/w): 0.1% BSA (15 µM), 0.1% lactic acid, 0.1% urea, 0.5% NaCl, pH 6.5. (**b**) Plots of the total SAXS intensity versus time at the position of the plug for experiments using natural human sweat (same conditions as in Fig. 1b, see “Methods” and Supplementary Information SI1 for further details) or artificial sweat containing BSA from previous data^21^. Both semi-logarithmic plots show that plug density increases exponentially with time during the densification stage. The difference of scattered intensities between natural and artificial sweat is linked to the higher concentration of protein in artificial sweat. (**c**) 3D reconstruction of plug from confocal microscopy images acquired in same conditions showing fluorescent aggregates located in the sweat channel, 30 min following contact. The artificial sweat contains 0.1% FITC-BSA. Dashed blue lines represent edges and contours of the T-junction channel. Data from b and c also appear in Figs. 1 and 4 of another recently submitted manuscript^25^.

Similar results, shown in Fig. 2a, were obtained using artificial sweat instead of natural sweat. Artificial sweat^21^ is a 0.5% NaCl aqueous solution containing 0.1% urea, 0.1% lactic acid and 0.1% BSA (15 µM) at pH 6.5. As for natural sweat, aggregates start to appear after a few minutes at a depth of 50–100 µm inside the sweat channel and the plugs get denser within 30 min. No clogging occurs in control experiments performed using artificial sweat without BSA. Given the similarity of the plugging profiles for natural and artificial sweat, the latter was thus considered as an appropriate model to study the clogging mechanism(s) of ACH.

It is worth noting that there is a lag time before the apparition of the first signs of aggregation inside the sweat channel: i.e. the first visible cloudy appearance starts after approximately 3 min following contact of the two fluids (see Fig. 2a). The structure of the gel tends to become denser within the next few minutes but does not seem to be completely homogeneous. The internal edges of the plug are forming on both sides up until 8 min, the rest of the channel being free from aggregates. From then on, the general shape of the plug does not evolve significantly, except by further apparent densification until the end of the experiment at approximately 30 min. Even after 30 min, the channel is not fully plugged as the device still maintains constant pressure delivering a reduced but positive sweat flow. This shows that the plug is either porous or that a stream of sweat flow can find a free duct through the gel. It is also particularly important to note that no aggregation is visible at the intersection of the two channels. This is a non-trivial observation since ACH polycations might have been expected to induce aggregation upon contact with proteins at the outlet of the sweat channel.

Densification can be studied by measuring the evolution of the total SAXS intensity, *I*, scattered by the plug formed with natural sweat (see Fig. 2b). The amount of matter forming the gel increases exponentially with time. This is similar to results described in our previous paper using artificial sweat^21^, thus confirming that our artificial sweat and natural sweat behave similarly. Insights into the structure of the plug through fitting of the data were not successful because the signal was too noisy for natural sweat reference. This is due to the low protein concentration in natural sweat and the low thickness of the sweat channel (50 µm).

**Figure 3:**
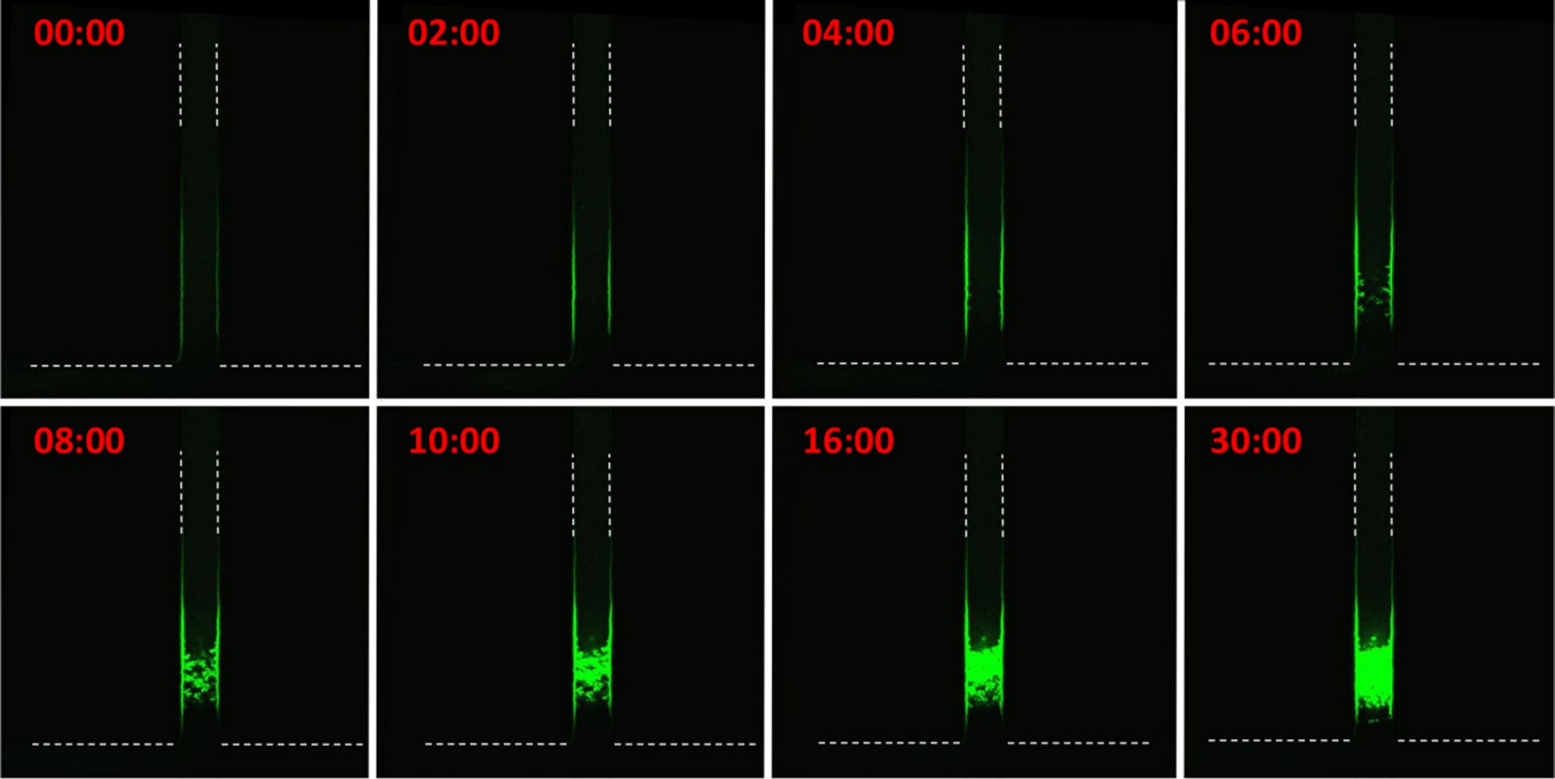
Confocal microscopy images obtained as optical sections located in the middle of the channel showing time-dependent apparition of fluorescent aggregates at 488 nm within 50 µm sweat channel at different times in min following contact of the two fluids using artificial sweat containing 0.1% FITC-BSA. Same flow conditions as in Fig. 2a caption. Dotted white lines delimit the contour of channel walls where no fluorescence is detected. Fluorescence aggregates appear in green.

Observing the plug formation using conventional optical microscopy is useful to study global parameters such as plug localization and shape. However, it does not give precise information related to the spatial structure of the plug. Therefore, we used a BSA-FITC conjugate instead of BSA to perform fluorescence confocal microscopy. BSA-FITC was dialyzed in order to remove traces of fluorescent small molecules such as fluorescein which could contribute to the aggregation because of their anionic nature. Figure 2c shows an example of 3D reconstruction that can be obtained using this technique. Such images are in complete agreement with the observations using conventional optical microscopy, but they do contain much more information due to optical sectioning at different depths inside the forming plug. Figure 3 shows optical longitudinal sections located in the middle of the sweat channel at different times. It can be seen that, shortly after filling channels with both fluids, a fluorescence is observed on the walls of the sweat channel suggesting adsorption of BSA-FITC conjugate on the walls of the channel. The fluorescence intensity continues to increase for a few minutes. Then, some “fluffy” low density fluorescent objects apparently attached to the walls start to grow and progressively densify while filling the space towards the centre of the channel. Images clearly suggest a heterogeneous nucleation from channel walls at a 50–100 µm depth from the sweat channel outlet. The adhesion of BSA-FITC conjugate all along the sweat channel walls is expected since PDMS is known to adsorb proteins^26^. However, fluorescence is not present beyond 200 µm from the outlet. This suggests that BSA-FITC conjugate interacts with ACH diffusing along the walls up to 200 µm, and not further.

We will report in a separate article more details revealing how the plug develops from the pore walls^25^.

### Physical chemistry of plug formation

**Figure 4:**
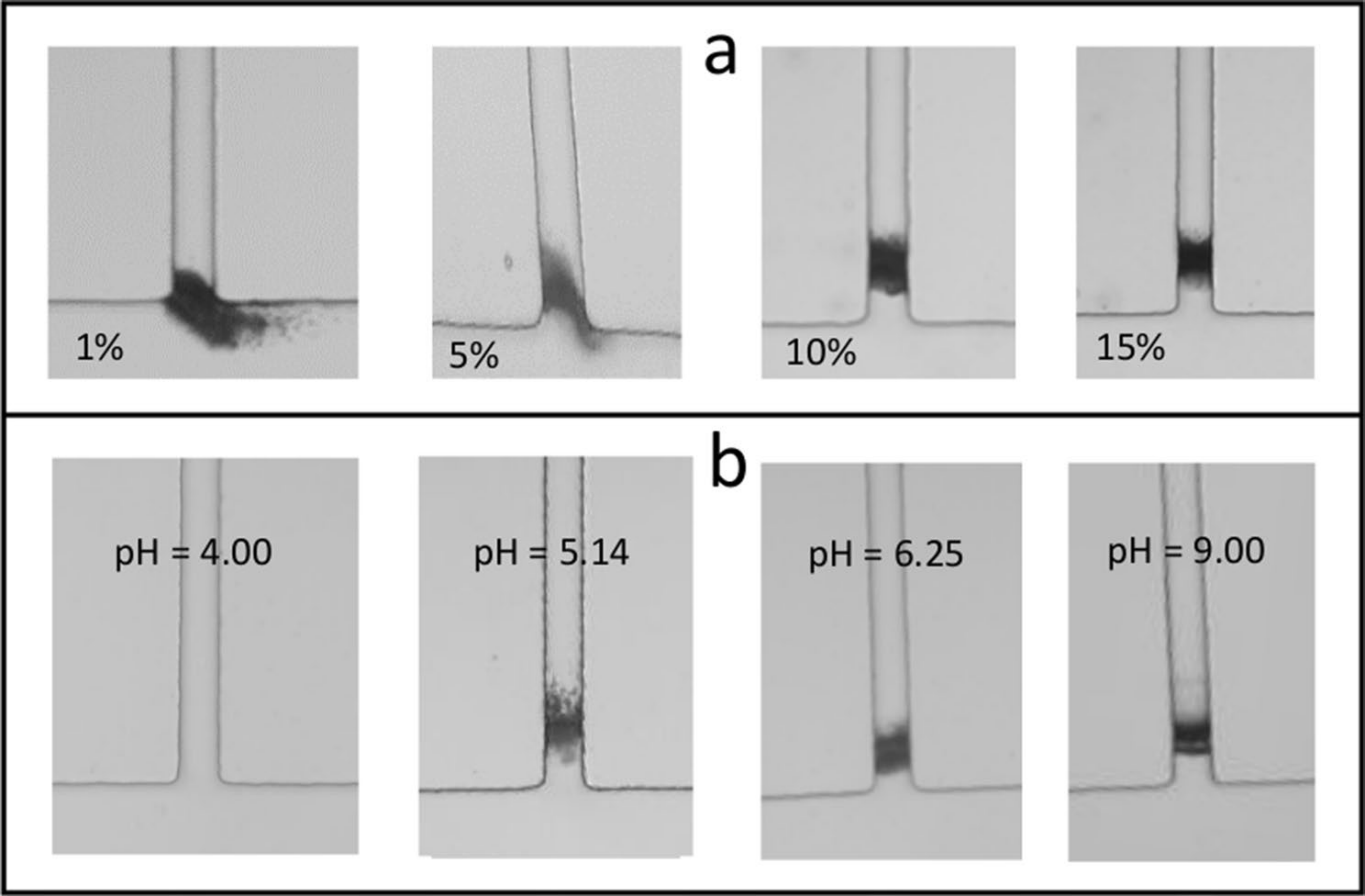
Pictures of plug formation within the sweat channel after 30 min using artificial sweat containing 0.1% BSA (15 µM). Same flow conditions as in Fig. 3 caption. (**a**) For aqueous solution of ACH at different concentrations from 1 to 15%. (**b**) Idem for different sweat pH from 4 to 9 (adjusted with ammonia) and an aqueous solution of ACH 15%.

In order to study the mechanisms involved in pore clogging, different experiments were conducted by varying: the ACH concentration, sweat pH and the composition. Figure 4a shows that plugs at 30 min obtained with 10% and 15% ACH are similar both in terms of location inside sweat channel, symmetrical shape and apparent density. However, at 5% ACH concentration, plug shape tends to be asymmetrical and slightly more superficial, i.e. the maximum density appears to be closer to the sweat channel outlet than at higher concentrations. The tilted orientation of the plug is the result of hydrodynamic forces on the aggregates. In the case of this superficial plug, it is likely that the ACH flow has a strong influence on the orientation. Part of the aggregates is located outside of the sweat channel, i.e. a small tail is carried outside the outlet by the sweat flow suggesting that the protein gel is soft and deformable. The same trend is even more pronounced at 1% ACH: most of the aggregate is located outside of the sweat channel with a bigger tail driven by sweat flow, although the same pressure controlled conditions are applied for all these experiments.

It is interesting to note that the ACH concentration range necessary to induce BSA aggregation in bulk solution is much lower compared to what is observed in microfluidic conditions. Indeed, when artificial sweat and ACH are mixed in bulk solution, flocculation only occurs in the range 0.01–1% ACH but not at higher concentrations. The likely reason for this discrepancy is that proteins are fully saturated by aluminium polycations at high ACH concentrations in bulk solution but not in microfluidic environment because of diffusion gradients. This point will appear more clearly under the light of numerical simulations studies described below.

Figure 4b illustrates the influence of the pH of the BSA artificial sweat in the range pH 4 to pH 9. Below the isoelectric point (IEP) of BSA (4.7), no plug is formed in the channel whereas above the IEP, aggregates are formed. In other words, plugging occurs only when BSA is negatively charged, which is in agreement with an aggregation mechanism resulting from an electrostatic interaction between aluminium polycations and negatively charged BSA. It is worth noting that the plugging pattern observed at pH 5.14, which is not far away from IEP, has a fluffier appearance than at higher pH which looks more densely packed. This could suggest weaker interactions between aluminium polycations and BSA at pH near IEP where the BSA’s negative charge is low compared to conditions at higher pH.

As already discussed in our previous paper^21^, BSA is not present in human sweat and it is therefore important to check whether proteins representative of human sweat behave like BSA. Human sweat indeed contains a variety of peptides and proteins (in the form of glycoproteins), including serum albumin, at a total concentration in the range of 0.05–0.08 g L^−1^, which corresponds to the low µM range^18,27^. In this perspective, experiments were performed with artificial sweat containing model glycoproteins, namely fetuin and mucin (see Supplementary Information SI2). In the case of BSA (66 kDa) and fetuin (64 kD), it corresponds to approximately 15 µM, whereas for mucin (640 kDa), it corresponds to 1.5 µM. In each case, below the isoelectric point of the concerned protein, no aggregation occurred. Beyond the IEP, the sweat channel is plugged and the kinetics of aggregation are of the same order compared to experiments with BSA. This is further evidence for the role of electrostatic interactions in the aggregation even when glycoproteins are involved. It also demonstrates the robustness of the microfluidic sweat flow model.

**Figure 5:**
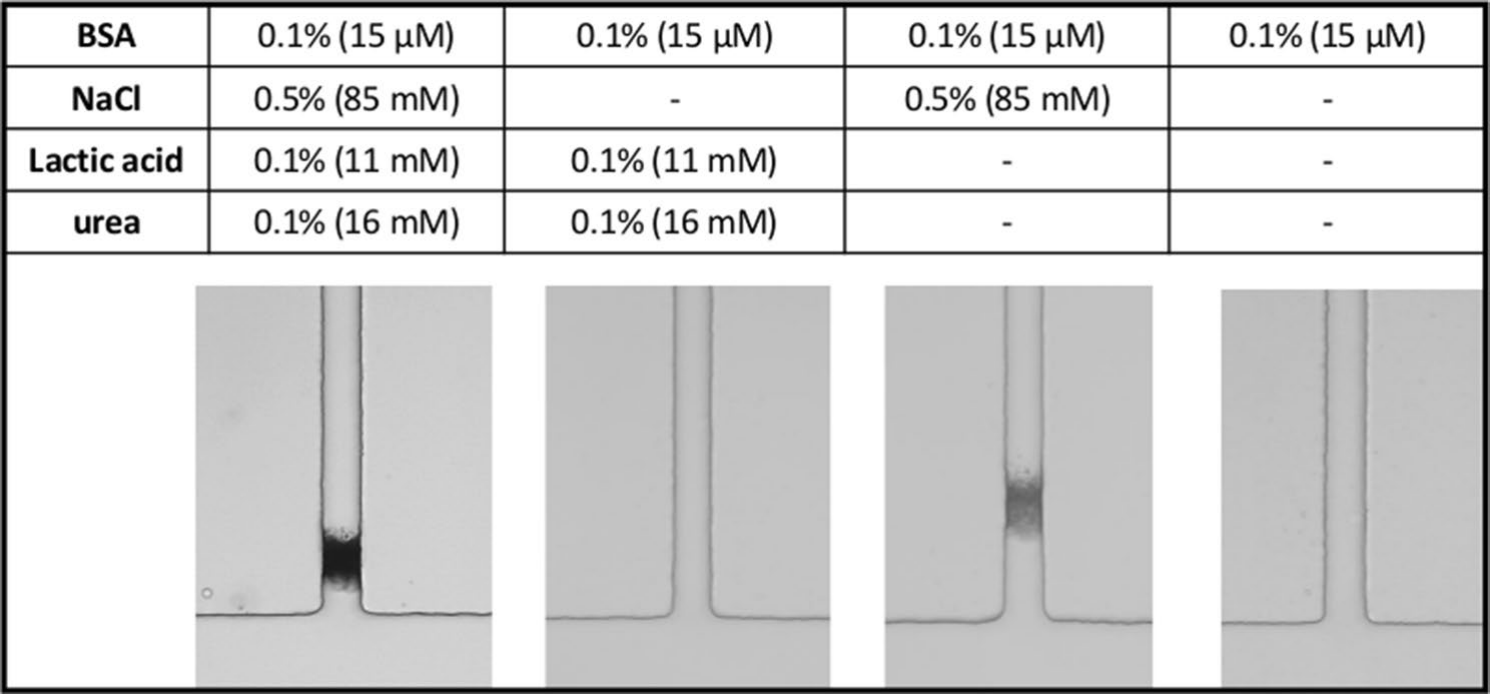
Pictures of plug formation within the sweat channel after 30 min. Same flow conditions as in Fig. 2 caption using artificial sweat of different compositions at pH 6.5. The respective compositions of the artificial sweats are shown in the Table above the pictures.

Results obtained with different compositions of artificial sweat are shown in Fig. 5. Images show that aggregation occurs only in the presence of NaCl since nothing happens after 30 min with artificial sweat containing either BSA alone or a combination of BSA, lactic acid and urea without NaCl. On the other hand, a comparison of the first image with the third image from the left on Fig. 5 shows that lactic acid and urea tend to increase plug density. Remarkably, the plug obtained without lactic acid and urea is located deeper in the sweat channel. It is also interesting to note that plugging with BSA/NaCl solution occurs at a faster rate than with full sweat since the first visible aggregation events appeared after approximately 1 min compared to 3 min for full sweat.

### Role of electrostatic interactions in plug formation

The absence of observed plugging for artificial sweat at pH below the IEP of model proteins (Fig. 4b, Fig. SI2) strongly supports a mechanism involving electrostatic interactions as the main driving force for sweat pore plugging. Indeed, BSA (IEP = 4.7) carries a distribution of negative charges on its surface due to a large number of glutamate and aspartate residues^28^, with a formal net charge of − 13 at pH 7. On the other hand, although the BSA-FITC conjugate bears a charge distribution slightly different from BSA^29^, aggregation occurs similarly (Fig. 3). The number of negative charges on proteins is of critical importance for their electrostatic interaction with polycations such as ε-Al_13_ and Al_30_, which have very dense and localized effective positive charges.

In the same spirit, the results obtained with fetuin are also of importance because fetuin is a glycoprotein—i.e. it contains oligosaccharide chains (glycans) covalently attached to amino acid side-chains—and therefore is structurally closer than BSA to proteins naturally present in sweat. Mucins are also heavily glycosylated proteins that are naturally present at the surface of the sweat duct epithelium. Their interaction with aluminium polycations is in agreement with their negative charge, corresponding to a large number of sialic acid residues that are deprotonated at sweat pH. Finally, stratum corneum keratins, which are insoluble proteins constitutive of the corneocyte surface at the entrance of sweat duct (acrosyringium), are also negatively charged at neutral pH since the IEP of keratin-10 and keratin-9 are 5.3 and 5.0, respectively^30^. Therefore, they could play a role in the nucleation or adhesion of aggregates at the surface of real sweat ducts.

The strength of electrostatic interactions between macromolecules also depends hugely on the variation of ionic strength. Indeed, ionic interactions affect BSA stability through electrostatic repulsion and attraction with polyelectrolytes^31^. The absence of aggregation when artificial sweat does not contain NaCl probably results from repulsion of BSA molecules whose charges are not screened (Fig. 5). On the other hand, in the presence of 0.5% NaCl (85 mM), which is representative of natural sweat, coulombic repulsions are screened and electrostatic attraction forces between BSA and aluminium polycations locally overwhelm the overall repulsions. The prompt apparition of the first aggregates in the absence of lactic acid and urea compared to full sweat may reflect another balance of the partially screened charge-charge interactions and would require additional studies to understand further.

Overall, our experimental results bring new insight into the mechanisms of sweat pore plugging by ACH. In particular, the results presented in the previous section are fully consistent with mechanisms involving electrostatic interactions between proteins and aluminium polycations, at specific locations in the sweat channel where both hydrodynamic and diffusive flows achieve sufficient protein and polycation concentrations with positive and negative electrical charges balancing. The aggregates grow by capturing proteins from the sweat flow, becoming denser and thus reducing the flow. However, further discussion is necessary to clarify these findings. For example, no general formula can be derived relating the location of the plug and the various physicochemical parameters used in the experiments. Below we detail the role of numerical simulations in understanding the mechanisms involved in pore plugging.

### Plug formation through numerical simulations

Controlled microfluidic experiments are essential to reveal the roles of physicochemical parameters in sweat pore plugging because they make possible to study individually the impact of factors such as the sweat composition or pH and ACH concentration as shown in Figs. 4 and 5. However, they do not fully help in understanding, at a molecular level, the mechanisms involved in plug formation. To understand further, it is necessary to know the collective movement of the molecules as a function of time, information which can be obtained by numerical simulation of the system. Below we present the results of our numerical simulations using our data obtained from the fore-described experiments. The numerical simulations have been specifically designed to study the dynamics of nucleation and growth of a plug inside a sweat channel.

**Figure 6:**
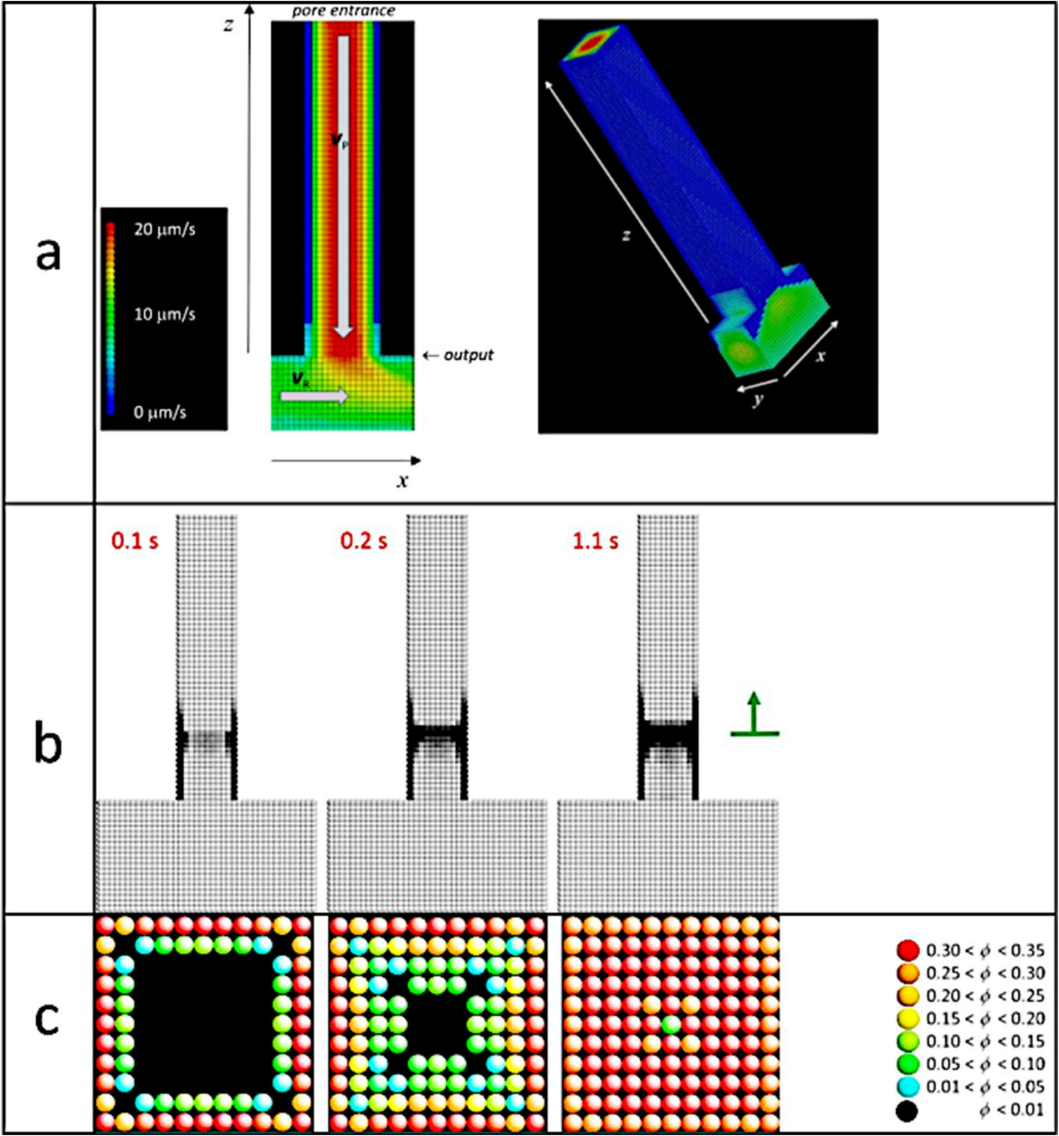
(**a**) Left: longitudinal section plane *y* = 0 of a pore of square section with the perpendicular ACH channel, used in the ATSIM3D numerical model. Pore outlet is marked with an arrow. A source of sweat proteins is placed at the pore entrance (top). Space is discretized in small elementary cubes of edge length 24 nm. In this example, the fluid velocity is *v*_P_ = −20 μm s^−1^ along the *z*-axis in the pore, and *v*_R_ = ±10 μm s^−1^ along the *x*-axis in the ACH channel. Colours correspond to the magnitudes of the velocity field (the colour map used for the velocities is on the left-hand side of the figure). Poiseuille flow is assumed in both channels. Right) Twisted view of the 3D simulated pore with flow inside. (**b**) Pictures of optical opacity during plug formation within the sweat channel at three different times in a 3D numerical simulation. Numerical parameters: space discretization = 24 nm; time discretization = 270 ns. System parameters: pore dimensions = 0.26 ×  0.26 ×  2.6 μm; ACH channel dimensions: 0.5  × 0.26  × 0.5 μm; pore flow velocity = 40 μm s^−1^ (top to bottom); ACH channel flow velocity = 10 μm s^−1^ (left to right). Chemical species: sweat protein concentration (source at the entrance of the pore) = 1 mM; sweat protein hydrodynamic radius = 3 nm and coordination number = 4 ; ACH molecule concentration (source upstream the ACH channel) = 1 mM; ACH hydrodynamic radius = 1 nm and coordination number = 4. Shades of grey follow: 1− *e* ^−*a m*^, with *m* is the total gel mass along the line of sight and *a* is a positive numerical coefficient (white = low density; black = high density). The green arrow on the right-hand side is a mark to point the cut plane. (**c**) Pictures of transverse sections in the middle of the plug (green arrow) inside the sweat channel, for the three times of the simulated system. The elementary cells are cubes in the simulations but they are represented here as spheres for better visual separation. False colours correspond to values of the local plug density, *ϕ*. The colour map is shown on the right-hand side of the figure.

The 3D numerical model (code name: ATSIM3D) involves solving finite-difference equations governing the movement of molecules inside the microfluidic device, within a fixed-grid method^32^. Physical time is used. In the simulations, sweat proteins are transported by laminar flow in the pore, and ACH molecules are transported by transversal laminar flow in the ACH channel. A visual sketch of the numerical system is shown in Fig. 6a.

The dynamic equations to be solved are:

- diffusion of the molecular species using the Fick diffusion equation^33^; every diffusing species (that is molecules or clusters of aggregated molecules) is characterized by its hydrodynamic radius
- advection of the molecular species through convection–diffusion equation^34^
- successive aggregation of the molecular species creating molecular clusters, a process ruled by Smoluchowski coalescence equations^35^.
- deposition of molecular species onto the pore walls or the gel. A sticking efficiency factor depending on the local sweat flow velocity was added to consider shear effects coming from the sweat flow in a simple way.

Full equations and additional information about the parameters are given in the Supplementary Information SI4. Because of computing-time constraints, the numerical code is limited in practice to pore diameter ≤ 1 µm, which is large enough to discuss the main mechanisms at work inside the pore. However, scaling the physical parameters is needed to do quantitative comparison with experimental data. This point will be presented below in “Scaling of the system during plug formation”.

Apart from the numerical parameters (dimensions of the microfluidic chip, space and time discretization), the relevant parameters of the numerical system are of two types. These are: (1) the physical parameters (flow rates and molecular concentrations in the sources); (2) the chemical parameters (hydrodynamic radius and coordination number of molecules). The initial time (*t = *0) is defined when the fluid carrying the sweat proteins in the pore just encounters the fluid with ACH molecules already flowing in the ACH channel.

### Plug formation at the channel walls

Opacity due to gel formation and to molecular clusters is shown for a simulated system in Fig. 6b at three different physical times. After a lag time, matter accumulates at a definite location inside the sweat channel, creating the plug. These numerical results can be compared to the experimental ones obtained with the BSA sweat microfluidic model shown in Fig. 2a.

**Figure 7:**
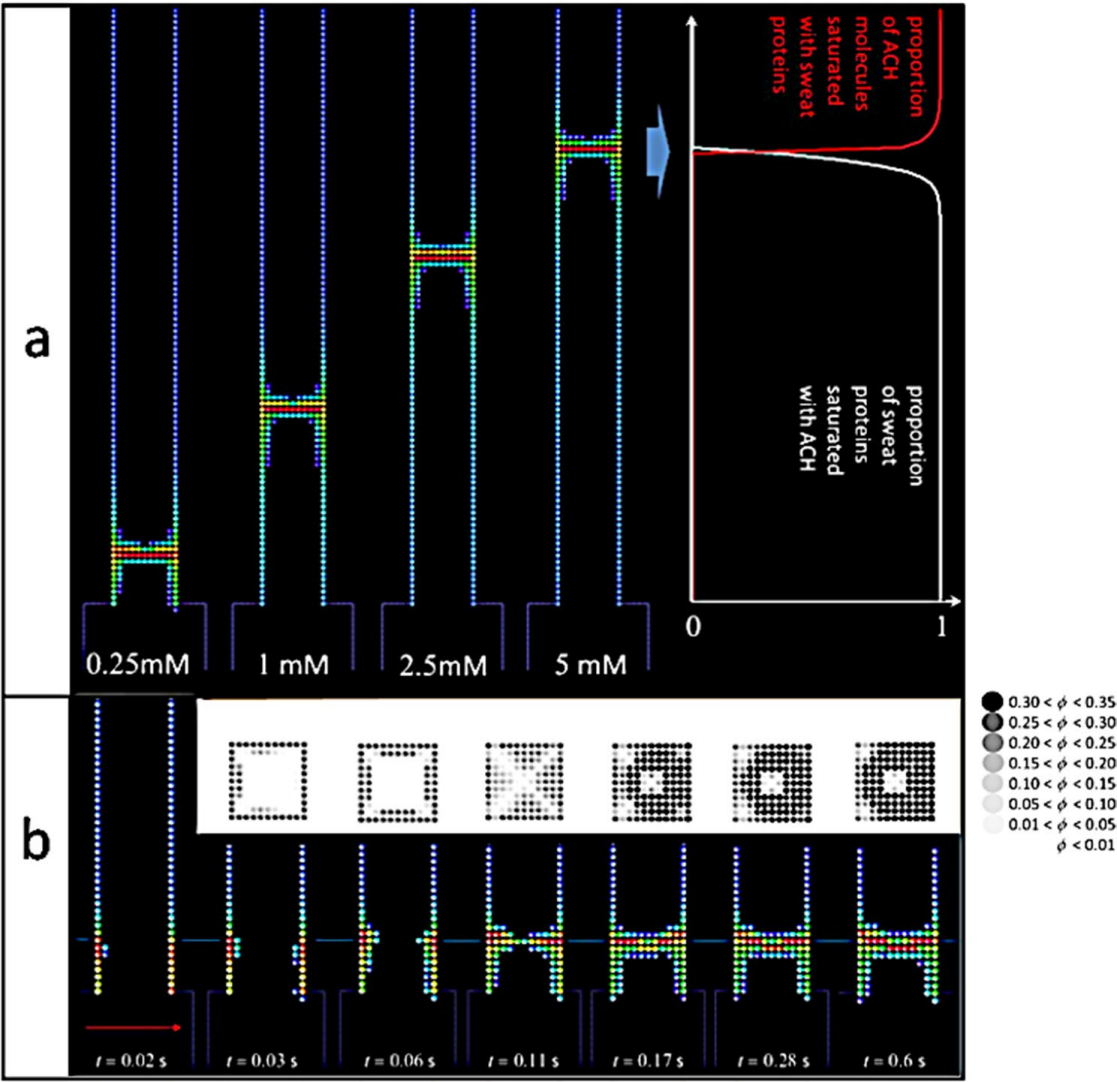
(**a**) Plug formation within the sweat channel in numerical 3D simulations of aqueous dispersion of sweat proteins at 1 mM, flowed at 20 μm s^−1^ (top to bottom), and aqueous solution of ACH molecules at four different concentrations *c*_ACH_ (0.25–5 mM), flowed at 10 μm s^−1^ (left to right). The numerical system is as in Fig. 6 and physical time is 2 s in the four cases. Pictures are sections *y* = 0 of the pore. False colours correspond to gel density (same colour scale as in Fig. 6c). The data plot shows, for the *c*_ACH_ = 5 mM case, the percentage of sweat proteins saturated with ACH molecules (in white colour) and of ACH molecules saturated with sweat proteins (in red) versus depth along the pore. Comparing to the picture for *c*_ACH_ = 5 mM, the conclusion is that plug forms in the region where molecules are not saturated. (**b**) Successive images of plug formation at increasing times (from left to right). Only the leftmost figure shows the whole length of the pore. Constituents are 3 nm sweat proteins at 1 mM, and 1 nm ACH molecule at 1.5 mM. Sweat flows in the pore from top to bottom at 100 μm s^−1^, that is a much larger than in Fig. 6. The red arrow (bottom left) represents the direction of the ACH flow at 10 μm s^−1^ inside the ACH channel. The six images in the top row are transversal sections of the pore at the corresponding times, at the constant depth marked by the horizontal blue line. Grey shades are used to visualize the structure of the gel (the grey map is shown on the right-hand side of the figure).

We note in particular that the channel walls are coated with matter before and after the plug, but not beyond about twice the depth of the plug. This indicates that ACH molecules either cannot go or cannot be active deeper than this distance. Full explanation of this fact requires an alternative result coming from the Fig. 7a below, that shows the influence of ACH concentration on the plug position. More specifically, Fig. 7a details the amount of sweat proteins saturated with ACH molecules (in white colour) and of ACH molecules saturated with sweat proteins (in red colour) as a function of depth inside the pore (see plot on the right-hand side of Fig. 7a). On that same figure, the corresponding plug structure is shown (in the longitudinal cut of the sweat channel) in false colours to the left of the plot (the plot corresponding to the system at *c*_ACH_ = 5 mM).

Plug formation occurs clearly in the narrow region where the diffusing molecules are essentially unsaturated, then capable of reacting with each other. Beyond a short distance deeper than the plug, the percentage of unsaturated ACH molecules drops to zero because of the number of active sweat proteins.

In “Results” section, we mentioned “*the general shape of the plug does not evolve significantly, except by further apparent densification until the end of the experiment*”. This point can be checked by numerical simulation by studying the evolution of the transversal cuts at a given depth where the plug appears. This is exemplified in Fig. 6c.

One can see in these figures that, as time goes by (time increasing from left to right), the plug is building up starting from the walls of the pore, then spreading gradually to the pore centre. On the final picture (right-hand picture), the centre is a green cell, that is a low density cell through which sweat can still flow. Plug is not homogeneous in the transversal plane, exhibiting high porosity around the axis of the pore. There is another, subtler, feature on this same picture: colours all around the centre are red (high density gel), while the layer closest to the walls are of orange colour, that is of smaller density than inside. This is the sign that gel densification is less effective near the walls than inside the pore. This behaviour will be explained in the next section.

Pictures in Fig. 6c are essentially symmetric with respect of the pore axis. However, in some cases, this symmetry is not realized. For example, if the concentration of ACH is too low or the sweat rate too high, the plug tends to form close to the pore outlet, and strong interaction with the flow makes the plug non-symmetric. This point was already noticed in the experimental pictures shown in Fig. 4a (we noticed: “*The tilted orientation of the plug is the result of hydrodynamic forces applied on the aggregates*”). Similar behaviour is seen in Fig. 7b corresponding to a large flow rate in the sweating pore. In this example, the structure of the plug exhibits three preferential free channels (in white colour on the pictures in the top of the figure) for the fluid to flow through, namely: the pore axis (where sweat can flow) and two corners of the pore, efficient for the ACH to diffuse upstream.

Before closing this subsection, one should note that the concentration of ACH used in the numerical simulations is different from the values used in the experiments. For example, 10% ACH (as seen on Fig. 4a) corresponds to 33 mM, while ACH concentration is equal to 1 mM in the numerical simulation shown in Fig. 7a. In the same way, times reported on Figs. 6b and 7b, are small compared to the experimental times shown in Fig. 2. Our purpose here is not to reproduce the experimental data, but to unveil the mechanisms leading to pore plugging. We will see later (formulae (3), (4)) that the data—such as plug position or gel time—have to be scaled by factors depending on the parameters, such as the size of the pore, the velocity of the flow etc. For example, the characteristic time, *τ*, is proportional to the diameter of the pore, then *τ* is naturally much smaller in the numerical simulations than in the real experiments.

### Scaling of the system during plug formation

The numerical code ATSIM3D is limited in practice to pore diameter ≤ 1 μm because of computing-time constraints. Even so, this code is helpful to analyse various mechanisms leading to the plug, though it is not designed to fit directly the experimental data. There are two ways to overcome this limitation.

1. The first option is to simplify the 3D numerical code in such a way that the simplified version is able to manage parameter values of the same order as the experimental ones. Such simplification is of course at the cost of a loss of information. Nevertheless, we investigated a 1D version of the code ATSIM3D, replacing the local volume fractions (depending on (*x*,*y*,*z*)) of the various molecular species by their transversal-averaged values (depending on the *z* coordinate only). Presence of the gel at the depth *z* results in a smaller radius, *a*(*z*), of the pore. In this model, the value of the pore radius is then: $a\left(z\right)= {a}_{0}\sqrt{1-\varphi (z)}$, in which $\varphi$(*z*) is the averaged volume fraction of the pore at that depth and *a*_0_ the initial pore radius. Such approximation for *a*(*z*) corresponds to a case where the gel is packed uniformly in a compact structure on the walls of the pore. Within such a model, the 3D Fick diffusion equation is replaced by the 1D Fick–Jacobs diffusion equation in a pore of variable section^36^. Even after adding the Smoluchowski terms, the final equations are much easier to solve numerically than in the 3D case. However, we lose all information related to the fine structure of the gel in the transversal planes. Such 1D numerical code (named ATSIM1D) is able to take into account pores of realistic diameters (20–30 μm). Details of the model are given in Supplementary Information SI5.
2. the other option is to compare smaller sized numerical simulations to real experiments knowing how the results (e.g. the plug position) scale with the parameters (e.g. the pore diameter). It can be done as follows. We assume that sweat protein propagation occurs as downstream advection with no diffusion. Assuming that gel forms in location where neither ACH molecules nor sweat proteins are saturated, one can state that the gel position, *z*_plug_, is located in the region where the local ACH concentration, *c*_ACH_(*z*), is approximately equal to the local sweat protein concentration, *c*_P_(*z*). If the ACH molecules diffuse along the pore walls, and the sweat protein concentration is essentially constant in that part of the pore, one finds (details in Supplementary Information SI6, equation (si12)):
  1 $${z}_{\mathrm{plug}}={z}_{0};\mathrm{ ln}\left(\frac{{\mathrm{C}}_{ACH}}{{\mathrm{C}}_{P}}\right) ;{z}_{0}=\sqrt{\frac{{d}_{o} {\Phi }_{w}}{{a}_{ACH}{v}_{P}}}$$

where Φ_w_ = *k*_B_*T*/(6π*η*)  = 0.24 μm^3^/s for water at ordinary temperature, *d*_o_ = 2*a*_0_ is the diameter of the pore, *v*_P_ is the mean velocity of sweat in the pore and *a*_ACH_ is the hydrodynamic radius of an ACH molecule. Note the slow logarithmic increase of the gel position with the input amount C_ACH  _of ACH molecules. The relation (1) leads also to a characteristic time, *τ*. Writing that *z*_0_^2^ ~ 2(Φ_w_/*a*_ACH_) *τ *(Brownian diffusion along the *z*-axis), one finds:

2 $$\tau = \frac{{d}_{o}}{2{v}_{P}}$$

For our experiments, Eq. (2) gives: *τ* ≈ 0.1 s, while we find *τ* ≈ 6 ms for the 3D numerical simulations shown in Fig. 7a. Equation (2) gives also the meaning of the characteristic time, *τ*, and of the length, *z*_0_, namely: *τ* is the time for the fluid to move by a length equal to the radius of the pore, while *z*_0_ is the distance an ACH molecule moves by diffusion during the time *τ*.

**Figure 8:**
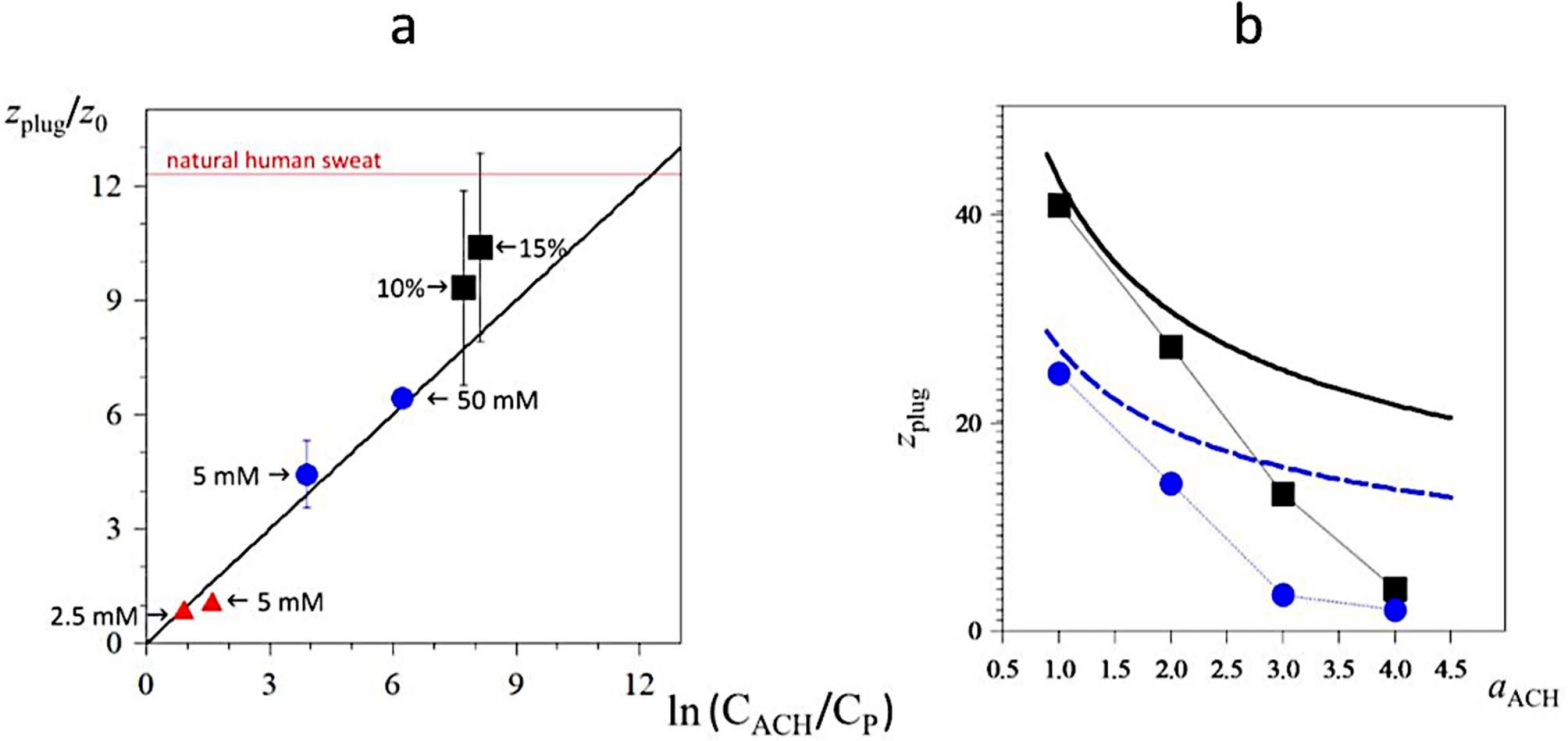
(**a**) Reduced plug position, *z*_plug_/*z*_0_, with the parameter *z*_0_ as given in (1), versus the natural logarithm of the sources ratio C_ACH_/C_P_ (the thick black line is the Eq. (1)). Black squares: experimental results shown on Fig. 4a for ACH concentrations 10% and 15%, and value of C_P_ = 15 μM (parameters used to calculate the value of *z*_0_: *d*_o_ = 59 μm (diameter of the disk of same area as a rectangle of edges 50 μm and 55 μm), *a*_ACH_ = 1 nm, *v*_P_ = 200 μm s^−1^). Blue circles: results from the 1D numerical code for ACH concentrations 50 mM and 5 mM, and C_P_ = 100 μM (parameters: *d*_o_ = 20 μm, *a*_ACH_ = 1 nm, *v*_P_ = 100 μm s^−1^). Red triangles: results shown on Fig. 7a, from the 3D numerical code for ACH concentrations 5 mM and 2.5 mM, and C_P_ = 1 mM (parameters: *d*_o_ = 0.26 μm, *a*_ACH_ = 1 nm, *v*_P_ = 20 μm s^−1^). The error bars correspond to the upper and lower extension of the plug in each experiment. (**b**) Mean gel localization inside sweat channel versus hydrodynamic radius of molecular aggregating species in the 1D numerical simulation model. Sizes of cylindrical sweat channel: *d*_o_ = 20 µm and 200 µm length. Sweat flows in the pore at constant 100 μm s^−1^ encountering aggregating species of coordinence 4 with different hydrodynamic radius at C_ACH_ = 5 mM (blue circles) or C_ACH_ = 50 mM (black squares). In this simulation, proteins (C_P_ = 0.1 mM) are considered as 3 nm particles with coordinence 5. The black thick curve is the Eq. (1) in the case of ACH concentration equal to 50 mM, and the dashed blue curve is for the 5 mM ACH concentration. There is no adjustable parameter. Agreement between Eq. (1) and 1D numerical simulations is excellent when plug forms well inside the pore, but poor when plug forms nearby pore output (because of the difficulty to define *z*_plug_ when part of the gel is located outside the pore).

With the relations (1) and (2) in hands, one can compare results coming from real experiments and from numerical simulations (which generally use much smaller pores than in microfluidics). Figure 8a shows some of the data (real and numerical experiments) with very different values of *d*_o_ (pore diameter), *v*_P_ (sweat velocity in the pore), and ACH concentration. The conclusion is that (1) and (2), though obtained using strong approximations, are accurate in all the cases, without the need for adjusting free parameter. The data from the microfluidic experiments are a little higher than the estimation (1); this might be due to the value of the hydrodynamic radius of the ACH molecule, taken here to be equal to 1 nm, and which is more plausibly equal to 0.65 nm. For the latter value, the two black squares are placed exactly on the black line. The thin red line on the top of the figure is the value of z_plug_/z_0_ in the real experiment shown on Fig. 1b, realized with natural human sweat. Since the concentration in ACH was 50 mM, one finds from (1) that the value of the concentration in proteins in the natural sweat should be ≈ 2.5 µM, which is a plausible value.

### Role of the antiperspirant molecule size in plug formation

Using relations (1) and (2), the mean depth of the gel attached to channel walls should increase as the square root of *d*/*a*_ACH_, that is the ratio between pore diameter and hydrodynamic radius of ACH molecule. This scaling would be a major result in the field, but it needs to be tested precisely, given the severe approximations used to derive (1) and (2). Such a check is not easy to do experimentally, but it is in principle feasible using numerical codes. Examples obtained using our 1D numerical code, are shown on Fig. 8b: mean positions of the plug are plotted against several values of *a*_ACH_. The pore is here a cylindrical channel of size (diameter × length): 20 × 200 μm, close to the physiological sweat pore dimensions. Moreover, the concentrations of aggregating species and protein were also chosen in the range of experimental conditions i.e. a large excess of ACH compared to sweat protein.

Two sets of representative conditions were used with an excess aggregating molecule (5 and 50 mM) and a low protein concentration (0.1 mM) in both cases. These values approach the experimental conditions. It is worth noting that with larger channels (20 µm) and highest concentration (50 mM), simulated mean gel depth is around 40 µm for smaller species (1 nm hydrodynamic species), thus approaching the experimental values (50–100 µm). Figure 8b confirms the trend already illustrated in Fig. 7a of the impact of concentration of aggregating species on the depth *i.e.* the higher the concentration in aggregating molecules, the higher the mean depth. It is also in agreement with the Eq. (1) obtained via a different route. However, comparison between the plug position in the 1D numerical simulations and formula (1) shows good agreement for the small values of *a*_ACH_, that is when plug forms well inside the pore, though there are clear systematic deviations when the plug forms close to the pore output (for the larger values of *a*_ACH_).

To conclude on the data shown on Fig. 8b, the simulations show that only highly concentrated species with a hydrodynamic radius smaller than 2–3 nm can significantly diffuse and build a plug localized inside sweat channel. This rule explains why cationic organic polymers of large molecular weight such as ionenes^37^ do not form plugs inside sweat channels (see Supplementary Information SI3), although they are known to aggregate polyanionic molecules or colloids by electrostatic mechanisms in bulk solution^38^.

It is also important to notice that not all aluminium salts behave like ACH in our model as for example potassium alum does not produce any plug (see Supplementary Information SI3). The absence of a plugging effect with potassium alum is consistent with the literature that describes that the presence of sulphate ions alters the pathway of aluminium polymerization to form charged polymeric materials^39^. In other words, Al_13_ and Al_30_ species are not generated from alum. This is likely to be linked to the coordination chemistry of bidentate sulphate bridging ligand compared to monodentate chloride counter ions in the case of ACH. This result strongly supports the role of aluminium polycations such as Al_30_ in plug formation, as already described by others^13,14^. Moreover, it also supports the relevance of our microfluidic model as a prerequisite for aluminium salts to have in-vivo antiperspirant efficacy since it is known that potassium alum has very low clinical antiperspirant effectiveness compared to ACH and functions more as a deodorant by having an antibacterial effect. ACH therefore seems unique because it brings together specific characteristics which are key performance drivers: small hydrodynamic radius and high net positive electric charge. It is also likely that the presence of different polycationic species in the ACH solution (from 1000 to more than 5000 Da) could cooperatively contribute to its efficacy. Moreover, the interaction of aluminium polycations on negatively charged sweat ducts walls is also likely to be an important factor to allow nucleation and growth of aggregates as mentioned above.

## Conclusion

The present work describes a series of investigations performed to reveal the mechanisms behind the antiperspirant action of aluminium salts. Two complementary tools were essentially used: (1) microfluidic device with artificial sweat was the key tool to carry out experiments with controlled parameters; (2) numerical simulations served to analyse at a microscopic level the behaviours seen in the experiments.

Experimental microfluidic results confirm, under physiological conditions, our previous observations^21^ that pore plugging occurs because aluminium polycations instigate sweat protein aggregation through electrostatic interactions. Confocal microscopy demonstrates that plugging starts on the walls of the sweat pore and extends towards the centre of the channel leading to progressive obstruction. SAXS data obtained with natural sweat reveals that the densification of the plug takes place in a short transition period between a lag time and a completion time, during which the amount of material constituting the plug grows exponentially with time.

The 3D numerical code simulates the complete microfluidic process, taking into account diffusion–advection and successive aggregation of molecular species. The simulations aid in explaining the influence of ACH concentration as well as the impact of flow conditions on the localization and general shape of the plug. They also highlight the porosity and heterogeneity of the plug in the early stage. The ACH molecules diffuse upstream along the channel walls enabling them to penetrate deep inside the pore. The first stage of the plug formation starts from the walls then spreads gradually to the channel centre. The second stage involves the rapid densification of the plug by abrupt entanglement. An analytical estimation (Eq. 1) of the position of the plug inside the sweat channel as a function of the physicochemical parameters is suggested. This equation is instructive. For example, the depth of the plug increases only logarithmically with the ACH concentration, then increasing that amount is in fact not so effective. On the other hand, the plug position is very sensitive to the sweat volume flow rate, so any additional process reducing this rate will result in a great improvement of the plug depth.

Overall, our experimental and numerical simulation data explains why large polycationic species are not as effective as ACH antiperspirants: only highly concentrated species with a hydrodynamic radius smaller than 2–3 nm can significantly diffuse and build a plug inside the sweat channel. Such findings explain why it has been so difficult in the past to find effective alternatives to aluminium salts. This work will hopefully help and inspire scientists to develop novel antiperspirant agents with improved performance.

## Methods

### Materials

Lactic acid, urea, sodium chloride, BSA, BSA-FITC conjugate, bovin fetuin and mucine (type III from porcine stomach) were purchased from Sigma-Aldrich. Aluminium Chlorohydrate (ACH) was purchased from Elementis (reference Chlorhydrol 50). Artificial sweat used has the following composition: 0.5% (w/w) NaCl, 0.1% lactic acid, 0.1% urea and 0.1% BSA at pH 6.5 (adjusted with ammonia). For fluorescence confocal microscopy monitoring, BSA-FITC conjugate was used instead of BSA. BSA-FITC conjugate was subjected to extensive dialysis in order to be sure that the solution was not contaminated by small fluorescein derivatives. Natural human sweat was collected from the armpit of volunteers after a sauna session and immediately kept frozen. All volunteers gave their written informed consent. The protocol complied with the Helsinki declaration and was approved by l’Oréal Research and Innovation Reading Committee.

### Microfluidic set up

PDMS chips were manufactured as previously reported^21^. Fluids were delivered using Fluigent pressure controllers MFCS-EX connected with 500 μm inner diameter PEEK (poly-ether-ether-ketone) tubing. A 20× objective lens mounted on an optical microscope equipped with a white light source was used for standard experiments to acquire images of T-junction at different times following contact between the two fluids. Sweat channel (55 µm high × 50 µm wide) were flowed at 0.6 nL s^−1^ at the beginning of the experiments, which corresponds to an average linear velocity is 218 µm.s^−1^, and then maintained at a constant pressure after few minutes. ACH flow in wider channel (55 µm × 400 µm) was flowed at 60 nL.s^−1^. At the beginning of the experiments, both sweat and ACH solutions were slowly flowed into empty channels up to apparition of the meniscus in both channels at a distance of approximately 50 µm from the T-junction. Images were then recorded for 30 min starting when both menisci get into contact. All experiments where the different parameters have been varied (concentrations, pH, compositions…) have been repeated at least three time.

### Confocal microscopy

Confocal microscopy images were acquired using a Leica DMI8 confocal laser scanning microscope with excitation wavelength at 488 nm. Optical sections were obtained every 5 µm steps inside sweat channel to access to plug 3D structure. Image J software was used to process the captured images.

### SAXS studies

T-junction X-rays resistant chips were designed and manufactured for SAXS studies. Sandwich-like chips were fabricated by the combination of photolithography, soft lithography and replication processes according to published procedures*.* In the photolithography step, a negative hard mould is obtained by patterning SU-8 with a dark field photomask. In the soft lithography step, the positive PDMS flexible moulds are produced. The chip is composed of one polyimide (PI) 13 μm thick and one layer of glass 100 μm thick assembled with a UV curable resin (Norland Optical Adhesive, NOA) spacer. To assemble the chip, a uniform layer of NOA is patterned with the flexible stamp on a PI film and partially cured under UV light. A glass cover slip is put in contact with the partially cured NOA, and then fully cured. The height of the channels reached 50 μm and the channels width was 50 and 200 μm (Sweat and ACH channels, respectively). SAXS measurements were performed at the coherent Small Angle X-ray Scattering (cSAXS) beamline at the Swiss Light Source (Paul Scherrer Institute, Switzerland). The X-ray beam was focused to 20 × 30 μm (vertical *x* horizontal) and the photon energy set on 11.7 keV (λ = 1 Å). Two-dimensional scattering patterns were collected by a Pilatus 2 M detector. The T-junction was placed in front of the focused beam at the position of the plug.

### Numerical models

Two different numerical codes have been developed to investigate the possible mechanisms leading to the pore clogging.

#### The 3D numerical model ATSIM3D

In this model, the device shape is similar to the design of the microfluidic experiment, namely: a long straight pore with rectangular section connected to a rectangular parallelepiped channel (see Fig. 6a). A regular grid made of cubes of side *a*_*l*_ serves as the discretization of space inside the pore and the ACH channel. Because of computer storage and computing-time restrictions, the typical pore diameter is limited to about 1 μm, and the pore length to about 4 μm. Aqueous dispersion of ACH molecules flows in the ACH channel at a velocity *v*_R_ perpendicular to the pore axis, while aqueous dispersion of sweat proteins flows inside the pore at a velocity *v*_P_ from the pore base into the ACH channel (see Fig. 6a). The flow is laminar^40^ since the Reynolds number is small (of order 10^−5^). The fluid exiting the pore through the orifice is a jet submerged in the fluid of the ACH channel, and the shape of that jet is a cone centred on the pore axis according to Hinze theory^41^. In the ACH channel, the velocity field of the mixed fluids is approximated as the sum of the velocity fields of each fluid. All the time, chemical species are dispersed in the fluid. They are: sweat proteins, ACH molecules, and molecular clusters that are combinations of proteins and ACH molecules. All clusters have volume up to the discretization cube. Within this rule, about 150 different sorts of molecular clusters are typically considered when *a*_*l*_ = 24 nm. Hydrodynamic radius (*a*_P_ and *a*_ACH_) of the protein and of the ACH molecule respectively are parameters of the model. The respective coordination numbers *κ*_P_ and *κ*_ACH_ are defined. It means that a protein molecule has *κ*_P_ pending bonds, while an ACH molecule has *κ*_ACH_ pending bonds. Molecular clusters are fractal aggregates of molecules with fractal dimension 2, in which each protein molecule share at least one protein bond with one ACH bond in a same cluster. Each molecular cluster is defined by its composition (number of protein molecules and number of ACH molecules) and its pending bonds (in number and nature). During a numerical simulation, the composition of the system evolves by diffusion, advection, aggregation and deposition (equations are written in Supplementary Information SI4) forming aggregates inside the sweat channel.

#### The 1D numerical model

The model considers the pore as a cylinder of variable circular section (diameter 2*r*(*z*,*t*)). The pore diameter changes due to the possible gel attached on the pore walls. In this case, the Reguera and Rubí equation^42^ is the basic equation describing the evolution of the diffusing species concentrations in a rectilinear pore of variable section, in the presence of a force field (here: the Stokes’ drag). The Smoluchowski’s equations are used as in the 3D model to quantify the aggregation events between the molecules. The equations are detailed in Supplementary Information SI5. A central approximation of this model is the estimation of the *z*-dependent equivalent diameter of the pore when gel is present. In the present version of this code, the gel is considered as if a compact material was uniformly distributed on the walls of the pore. Then, the equivalent diameter of the pore at the altitude *z* can be calculated from the concentration of the gel at that altitude. The equivalent pore diameter is required to be used in the Reguera and Rubí equation. Because the model is 1D (the dimension of the axis of the pore), the corresponding simulations are much faster than for the 3D model. Then, for a same amount of computing time, one can know the evolution of the gel and the various molecular concentrations as functions of the physical time, for numerical systems with realistic length scales (e.g. pore diameter of a few tens micrometers), over physical times of a few minutes. However, the 1D model cannot give precise information about the structure of the plug in transversal planes.

## Supplementary Information

Supplementary Information

## Data Availability

The datasets generated and/or analysed during this study may be made available by the corresponding author upon reasonable request.
