# Supplementary material for: Real time observation of the interaction between aluminium salts and sweat under microfluidic conditions
Source: Sci Rep. 2021 Mar 18;11:6376. doi: 10.1038/s41598-021-85691-8 (PMC7973555; doi:10.1038/s41598-021-85691-8)
Supplement: Supplementary file 1 — Supplementary Information [file 41598_2021_85691_MOESM1_ESM.pdf]

Supplementary Information

Real time observation of the interaction between aluminium salts and sweat under microfluidic conditions

Yasine Sakhawoth[2], Jules Dupire[1], Fabien Leonforte[1], Marion Chardon[1], Fabrice Monti[2], Patrick Tabeling[2], Bernard Cabane[4], Robert Botet[3*] & Jean-Baptiste Galey[1*]

[1] L'Oréal Recherche & Innovation, 1 avenue Eugène Schueller, 93600 Aulnay-sous-Bois, France

[2] IPGG, MMN, 6 Rue Jean Calvin, 75005 Paris, France

[3] Université Paris-Saclay, CNRS, Laboratoire de Physique des Solides, UMR8502, 91405, Orsay, France

[4] LCMD, CNRS UMR8231, ESPCI, 10 rue Vauquelin, 75231 Paris cedex 05, France

* Corresponding authors

SI1: SAXS data obtained with natural sweat

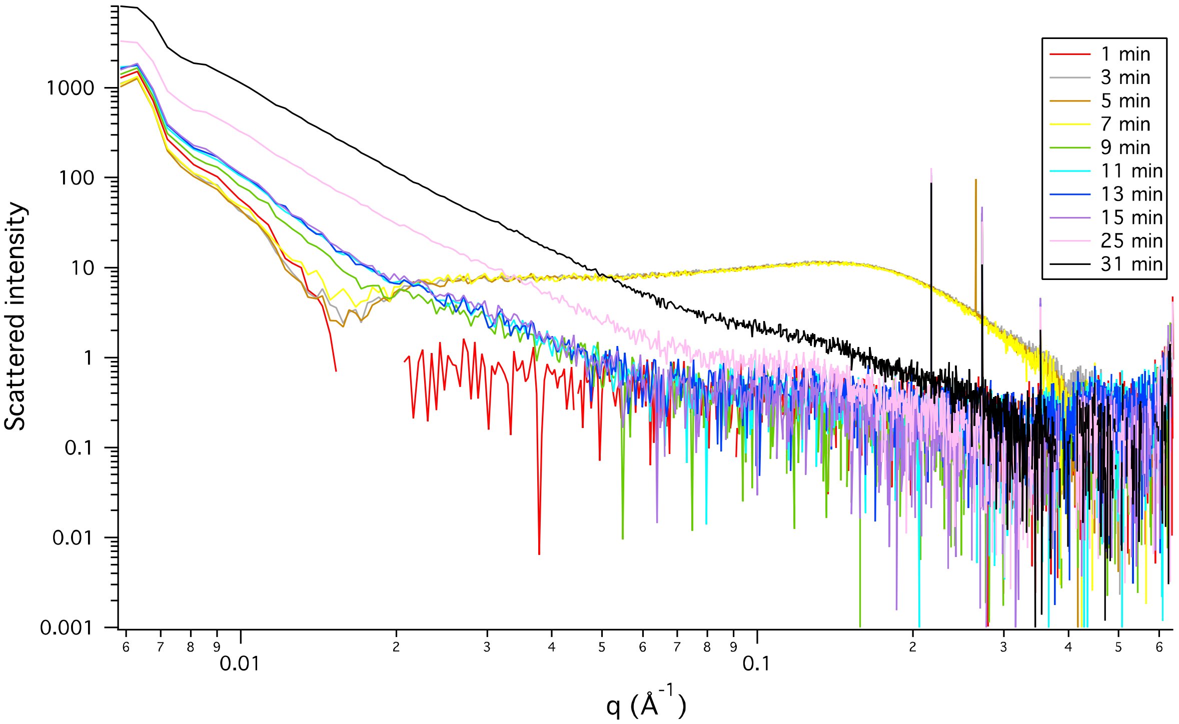

Figure SI1A. Small-angle X-ray scattering (SAXS) intensity as a function of wave vector q at the entrance of sweat channel in NOA microfluidic chip at different time following contact of natural sweat and 15% ACH solution. Scattered intensity of natural sweat is at level of the background at the beginning of the experiment. At low q-range, scattered intensity of protein from natural sweat increased progressively. At early times, diffusion of ACH in sweat channel is revealed by the presence of a shoulder at high q-range which disappears after 9 min. During previous SAXS experiment, BSA 1% was used in order to improve signal quality.

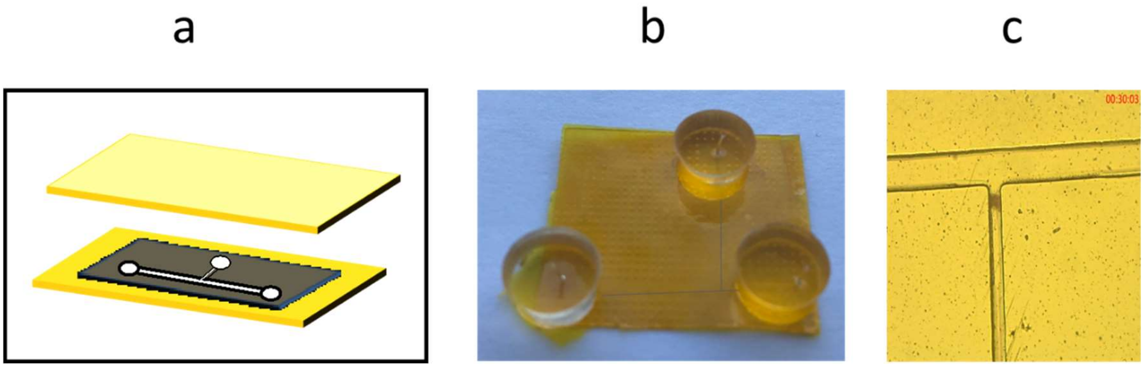

Figure SI1B. a. Schematic diagram of the SAXS microfluidic Kapton / NOA / Kapton chip used for the scattering experiments. b. Photograph of finally assembled chip with cylindrical PDMS plugs for tube fitting. c. Photograph of the plug formed within the sweat channel after 30 min using 15% ACH.

SI2: Plugging patterns obtained with fetuin and mucin

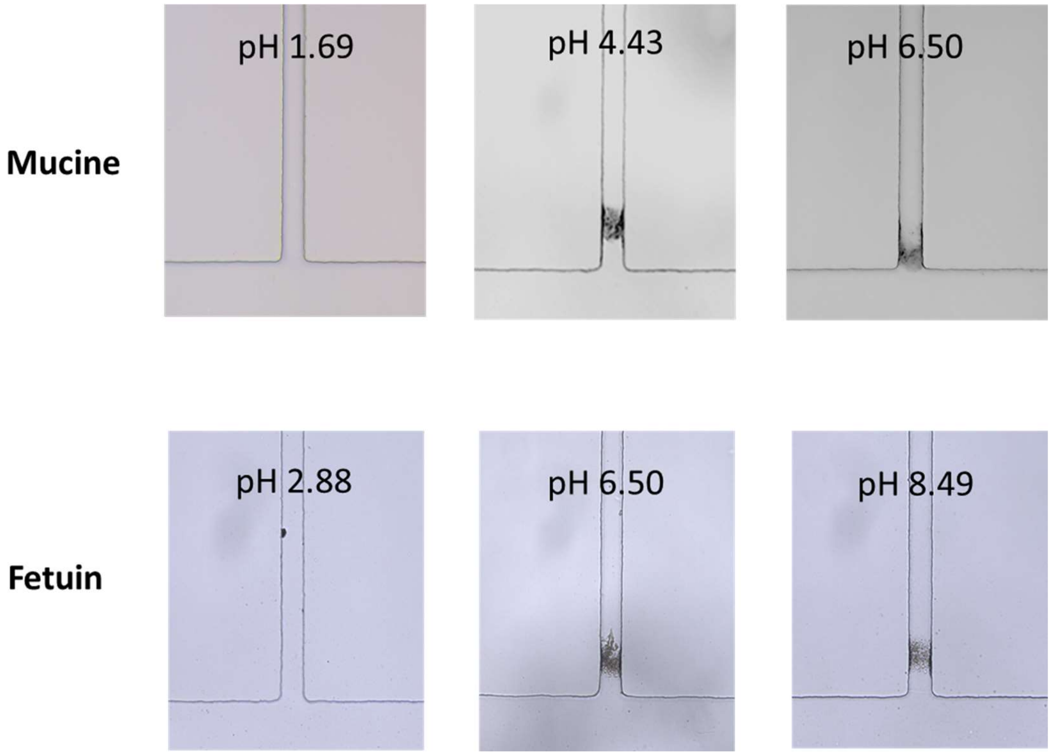

Fig SI2: Pictures of plug formation within the sweat channel using artificial sweat containing either 0.1% fetuin (IEP=4) or mucin (IEP=2) instead of BSA at different pH, flowed at 0.6 nL s^{-1} at the beginning of the experiment and maintained at a constant pressure in the sweat channel and an aqueous solution of ACH 15% (wt%) flowed at 60 nL s^{-1} in the ACH channel, demonstrating that plugging occurs only when proteins are negatively charged.

SI3: Plugging patterns obtained with other cationic species

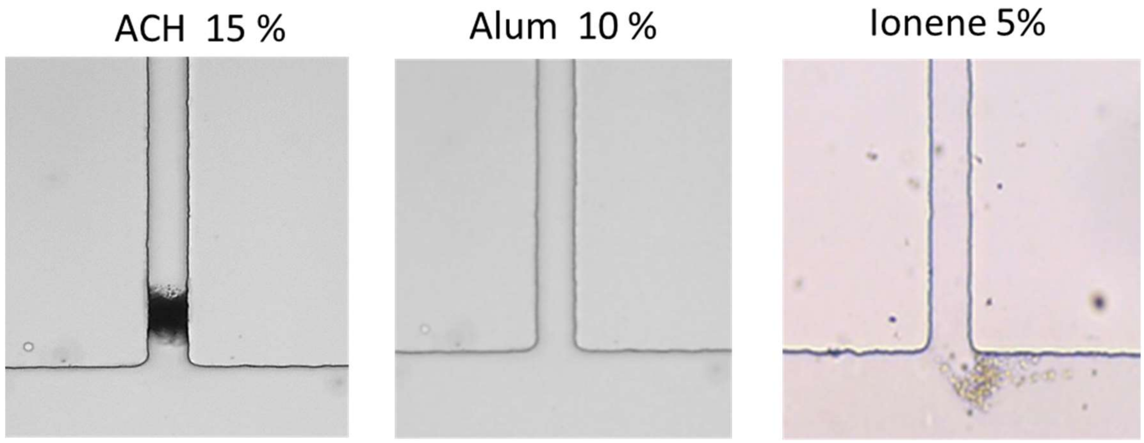

Figure SI3. Pictures of plug formation within the sweat channel after 30 min. Same flow conditions as in Fig. 2 caption using BSA artificial sweat and aqueous solutions (w/w) of 15% ACH, 10% potassium alum, or 5% ionene.

It can be seen from Fig. SI3 that potassium alum ($KAl(SO_4)_2$), which is a sulphate salt of aluminium, does not induce any plug or aggregate at 10%, in contrast to ACH. These results

are consistent with the literature which describes how the presence of sulphate ions alters the pathway of aluminium polymerization to form polymeric and solid materials[1].

On the other hand, the 3,3-ionene polyelectrolyte tested did not show any aggregation inside the sweat channel. Some aggregation can be seen outside of the sweat channel, but nothing appears inside. Ionenes are cationic polymers composed of quaternary ammonium centres as part of the main hydrocarbon chain, separated by a predefined number of methylene (CH_2) spacer units. 3,3-ionene used for these experiments[2] has a chain length around 100 nm and a mean molecular weight of 20,000 Da.

SI4: Equations used in the numerical code ATSIM3D

The numerical code ATSIM3D uses space discretization with space step a_l = 24 nm. Within the discretized space, the microfluidic device is defined precisely, though of smaller dimensions to save storage memory. At each time of a numerical simulation, the local number concentrations c_k of all the species k are known at any place of the system. The species are: sweat protein, ACH molecule, and all the molecular clusters (combinations of ACH molecules and sweat proteins consistent with the coordination number of every molecule) of hydrodynamic radius not larger than a_l. The hydrodynamic radius of each molecular cluster is calculated with formula[3]:

$$R_h \propto \left(N_P a_P^{d_f} + N_{ACH} a_{ACH}^{d_f}\right)^{1/d_f} \qquad \text{[si1]}$$

that is under the assumption that the cluster is fractal of fractal dimension d_f. According to Jullien and Botet[4], we can take: $d_f = 2$. All the molecular species move and react according to the following laws:

i. species diffusion. The diffusion equation[5] for the species k in both channels is:

$$\left.\frac{\partial c_k}{\partial t}\right|_{diffusion} = \frac{\Phi_w}{a_k}\nabla^2 c_k \qquad \text{[si2]}$$

in which $c_k(x,y,z,t)$ is the number concentration of the species k of hydrodynamic radius a_k at time t, in the discretized cube located in (x, y, z). The coefficient Φ_w / a_k is the Stokes-Einstein diffusion constant of species k, with $\Phi_w = k_B T/(6\pi\eta)$ related to dynamic viscosity of water, η, at temperature T.

ii. species advection by fluid motion[6]. If $\boldsymbol{v}$ denotes the fluid velocity vector at location (x, y, z):

$$\left.\frac{\partial c_k}{\partial t}\right|_{advection} = -\nabla(\boldsymbol{v} c_k) \qquad \text{[si3]}$$

iii. aggregation between molecular species[7]. The Smoluchowski coalescence equation holds, namely:

$$\left.\frac{\partial c_k}{\partial t}\right|_{aggregation} = \frac{1}{2}\sum_{i+j=k} K_{ij} c_i c_j - \sum_i K_{ik} c_i c_k \qquad \text{[si4]}$$

in which K_{ij} is the probability per unit of time of combining a cluster of species i and a cluster of species j to form a cluster of species k. The kernel K_{ij} is the product of the Brownian kernel and of the probability that a pending bond of the cluster i combines with a pending bond of the cluster j during a hit.

iv. species <u>deposition</u> on the gel:

$$\left.\frac{\partial c_k}{\partial t}\right|_{deposition} = -e^{-v/v_k} K_{gk} c_g c_k \qquad [si5]$$

in which K_{gk} is the probability per unit of time of combining a cluster of species k with the gel (c_g is the local gel concentration). The kernel K_{gk} is defined in the same way as the quantities K_{ij} above, with the gel as a motionless cluster of molecules. In [si5], the exponential term, exp($-v/v_k$), is the probability for the deposition to be effective onto the gel. Indeed, shear effects due to the sweat flow can prevent deposition, and, in a crude approximation, the exponent of this exponential term is minus the ratio between the work of the Stokes force over the diameter of the molecule k, and the energy, E_b, of a bond linking a sweat protein to an ACH molecule. Within this approximation, v is the local sweat velocity and $v_k = E_b/(12\pi\eta\, a_k^2)$ is a characteristic velocity, with η the viscosity of the sweat. For typical values of the parameters, the value of v_k is of order 1 m/s, and the effect of the shear is essentially a limited slowdown of the gel growth (without sensible effect on the shape or the location of the gel).

According to [si5], the gel grows according to the equation:

$$\frac{\partial c_g}{\partial t} = \sum_k e^{-v/v_k} K_{gk} c_g c_k \qquad [si6]$$

The kinetic equation for the species k writes in full:

$$\frac{\partial c_k}{\partial t} = \left.\frac{\partial c_k}{\partial t}\right|_{diffusion} + \left.\frac{\partial c_k}{\partial t}\right|_{advection} + \left.\frac{\partial c_k}{\partial t}\right|_{aggregation} + \left.\frac{\partial c_k}{\partial t}\right|_{deposition} \qquad [si7]$$

It corresponds typically to 150 coupled differential nonlinear equations. The equations are solved by forward Euler method because the equations [si8] are essentially parabolic, then exhibit good numerical stability. Numerical stability is dynamically checked during a simulation.

Various boundary and initial conditions can be used, and the final system state does not seem to depend much of the definite initial conditions. In the present work, we focus on the following conditions that correspond to the case where antiperspirant is applied while sweating process is active (this is similar to the experimental microfluidic case):

- initial conditions: $c_P(x,y,z,0) = C_P$ is realized inside the whole pore, and $c_{ACH}(x,y,z,0) = C_{ACH}$ in the whole ACH channel.
- boundary conditions: protein concentration is constant $= C_P$ at the pore output z_{max} (see Fig. 12), and ACH concentration is constant at the entrance of the ACH channel

(that is: $c_{ACH}(x_{min},y,z,t) = C_{ACH}$ for x_{min} = position of the ACH channel entrance, left part of Fig. 12).

SI5: 1D numerical model ATSIM1D

The numerical code ATSIM1D is a simplified variant of the 3D ATSIM3D code, in which the concentrations of every chemical species are averaged over transversal planes.

As we have no information (due to the averaging process) on the spatial distribution of gel within a given section, the local porosity of the gel is replaced by $(a/a_0)^2$, in which a_0 is the initial radius of the pore and $a(z,t)$ the effective radius of the pore at the depth z at time t. In other words, this effective radius is the radius of the pore as if all the matter composing the gel formed a dense layer all around the pore surface. The section, $S(z)$, of the pore at the depth z is then simply: $S(z) = \pi a^2$.

That way, the equations governing the evolution of these averaged concentrations are the same as in the 3D case (the equations [si4], [si5], [si6], [si7]), except the advection and diffusion equations [si1] and [si2], which must be replaced by the Fick-Jacobs equation[8]:

$$\left.\frac{\partial c_k}{\partial t}\right|_{Fick-Jacob} = \frac{\Phi_w}{a_k}\frac{\partial}{\partial z}\left(\frac{1}{\sqrt{1+a'^2}}\left[\frac{\partial c_k}{\partial z} - \left(\frac{2a'}{a} + \frac{1}{\Lambda}\right)c_k\right]\right) \qquad \text{[si9]}$$

in which the characteristic length $\Lambda = S(z)\,\Phi/(Q\,a_k)$ if the process occurs at constant volume flow rate Q, or $\Lambda = 8\pi\eta\,L\,\Phi_w/(a_k\,S(z)\,\Delta P)$ if it occurs at constant pressure gradient $\Delta P/L$. The notation a' represents the derivative of the effective radius a with respect to the depth z inside the pore. The same boundary conditions are used as in the 3D case.

This 1D numerical code can manage much bigger pore sizes than ATSIM3D, because it deals with two variables (z and t) instead of four (x, y, z and t). However, one has to be aware that the main approximation of ATSIM1D is serious: the sections of the pore with gel (gel volume fraction: $\varphi(z,t)$) are replaced by sections of variable effective radius $a(z,t) = a_0\sqrt{1-\varphi(z,t)}$, then the averaged gel volume fraction is correct but the distribution of the sweat flow is strongly approximated.

SI6: Approximation of gel position in the sweating pore

Inside the pore, sweat proteins and ACH molecules do not move through the same mechanism: proteins are carried by the sweat flowing in the pore, while movement of ACH molecules is essentially diffusive and hindered by sweat flow. Therefore, the concentration, c_P, in sweat proteins is approximately a constant, C_P all inside the pore before the gel position (because the flow feeds it at a constant rate), while the profile of the ACH concentration, c_{ACH}, is decreasing fast with the depth, z, in the pore. We then consider the following simplified problem in which ACH molecules diffuse along the pore walls while sweat protein concentration remains constant. These approximations allow to obtain a crude estimation of the position where both concentrations balance (that is the active region where plug can form).

The pore section is here circular of radius a_0. The sweat flows downward the z-direction of the pore at the average velocity: $v_P > 0$. The fluid velocity at the transversal distance $x a_0$ (with $0 \leq x \leq 1$) from pore axis is Poiseuille-type: $v = 2v_P\,(1-x^2)$. In places where the fluid velocity is small (that is $x \approx 1$), ACH molecules can diffuse upstream according to equations

[si2] and [si3] above. More precisely, Φ_w/a_{ACH} being the ACH molecule diffusion coefficient, transversal diffusion is dominant against removal by flow when: $((1-x)a_0)^2 < (\Phi_w/a_{ACH})((1-x)a_0/v)$. The largest value of x defining such diffusing channel for the ACH molecules is given by the equation:

$$(1-x)^2(1+x) = \frac{\Phi_w}{d_o\, a_{ACH} v_P} \qquad [si10]$$

where $d_o = 2a_0$ is the diameter of the pore. In most of the cases, the value of x is indeed close to 1 (that is the ACH molecules diffuse essentially along the pore wall), then:

$$1-x \approx \sqrt{\frac{\Phi_w}{2d_o\, a_{ACH} v_P}} \qquad [si11]$$

In the ring domain between $(1-x)\, a_0$ and a_0, diffusion-advection of the ACH molecules is ruled by the equation:

$$\frac{\partial c_{ACH}}{\partial t} = \frac{2\Phi_w}{a_{ACH}} \frac{\partial^2 c_{ACH}}{\partial z^2} + \langle v \rangle \frac{\partial c_{ACH}}{\partial z} \qquad [si12]$$

in which $<v> = v_P(1-x^2)$ is the average fluid velocity in the ring. The stationary solution of [si12], using the value of x given by [si9], is:

$$c_{ACH}(z) = \mathrm{C}_{ACH}\, e^{-z/z_0} \qquad [si13]$$

with:

$$z_0 = \sqrt{\frac{d_o\, \Phi_w}{a_{ACH} v_P}} \qquad [si14]$$

Plug can form in the region where neither ACH molecules nor sweat proteins are totally saturated. This region is characterized by the condition: $c_{ACH} \sim c_P$ (ACH molecule saturation occurs when $c_P > \kappa_{ACH}\, c_{ACH}$, and sweat proteins saturation when $c_{ACH} > \kappa_P\, c_P$, with typical coordination numbers κ_{ACH}, κ_P of order 3 to 5). Therefore, the position z_{plug} of the gel is estimated to be:

$$z_{plug} = \sqrt{\frac{d_o\, \Phi_w}{a_{ACH} v_P}} \ln\left(\frac{\mathrm{C}_{ACH}}{\mathrm{C}_P}\right) \qquad [si13]$$

References:

1. Perry, C.C. & Shafran K.L, The systematic study of aluminium speciation in medium concentrated aqueous solutions. J. Inorg. Biochem. 87, 115-124 (2001).

2. Sakhawoth, Y., Michot, L., Levitz, P., Rollet, A.L., Sirieix-Plenet, J., Hermida Merino, D. & Malikova, N. Aggregation of Plate-like Colloids Induced by Charged Polymer

Chains: Organization at the Nanometer Scale Tuned by Polymer Charge Density. Langmuir 33, 10937–10946 (2019).

3. Wiltzius, P. Hydrodynamic Behavior of Fractal Aggregates. *Phys. Rev. Lett.* **58**, 710 (1987).

4. Jullien, R. & Botet, R. *Aggregation and Fractal Aggregates* (World Scientific, Singapore, 1987).

5. Paul, A., Laurila, T, Vuorinen V. & Divinski, S.V. *Thermodynamics, Diffusion and the Kirkendall Effect in Solids* (Springer Cham, 2014).

6. Bennett, T.D. *Transport by Advection and Diffusion: Momentum, Heat and Mass Transfer*, (John Wiley and Sons, NJ 2012).

7. Galina, H. & Lechowicz, J.B. Mean-Field Kinetic Modeling of Polymerization: The Smoluchowski Coagulation Equation, In: *Advances in Polymer Science* **137**, 135-172, (V. Springer Berlin Heidelberg 1998).

8. Burada, P.S., Schmid, G., Ruguera, D., Rubi J.M. & Hänggi, P. Biased diffusion in confined media: Test of the Fick-Jacobs approximation and validity criteria. *Phys. Rev. E* **75**, 051111-1-8 (2007).
